# Circular transcriptome sequencing of the middle silk gland and posterior silk gland in the Bombyx mori

**DOI:** 10.1016/j.dib.2017.10.028

**Published:** 2017-10-18

**Authors:** Huaiyan Gan, Tieshan Feng, Yuqian Wu, Chun Liu, Qingyou Xia, Tingcai Cheng

**Affiliations:** State Key Laboratory of Silkworm Genome Biology, Southwest University, Chongqing 400716, China

**Keywords:** CircRNA-seq, Circular RNA sequencing, MSG, Middle silk gland, PSG, Posterior silk gland, *B.mori*, *Bombyx mori*, CircRNA-seq, Bombyx mori, MSG, PSG

## Abstract

Circular transcriptome sequencing of the middle silk gland (MSG) and posterior silk gland (PSG) in the *Bombyx mori* are presented. The middle silk gland and posterior silk gland were collected from the third day of fifth-instar *B.mori* larvae. The circular RNAs enriched by using RNase R to degrade the linear RNA molecules, and circular RNA sequencing (circRNA-seq) was performed using an Illumina Hiseq. 2500 sequencing platform. Samples are described in the SRA portal (SRP100385) and FASTQ files have been deposited in Sequence Read Archive (accession numbers: SRX2577343 and SRX2577342). The interpretation of these data is presented in the following research article: “Identification of circular RNA in the *Bombyx mori* silk gland” [1] (Gan et al., 2017).

**Specifications Table**

**Table:** 

Subject area	Biology, entomology
More specific subject area	Next generation sequencing
Type of data	Text file
How data was acquired	High throughput circRNA sequencing
Data format	Raw
Experimental factors	The MSG and PSG from day-3 fifth-instar larvae were dissected and collected,and the total RNAs were treated using RNase R
Experimental features	circular RNAs enriched by using RNase R treated total RNA
Data source location	Chongqing, China
Data accessibility	Full data sets have been submitted to NCBI Sequencing Read Archive (SRA, https://www.ncbi.nlm.nih.gov/sra) under accession number SRX2577343 and SRX2577342. Data are publicly available.

**Value of the data**

1. The data can be used to explore the circular RNAs that exists in the MSG and PSG of the Bombyx mori.
2. The data can be used to understand the feature of circular RNAs in the Bombyx mori.
3. The data can be used to analysis the expression pattern of circular RNAs in the MSG and PSG.

## 1 Data

Data provided in this article correspond to the FASTQ files obtained after circular RNA sequencing of the MSG and PSG in the Bombyx mori [1].

## 2 Experimental design, materials and methods

### 2.1 Sample collection

B. mori strain Dazao was obtained from the State Key Laboratory of Silkworm Genome Biology,Chongqing, China. Larvae were fed with mulberry leaves under normal conditions. The MSG and PSG from day-3 fifth-instar larvae were dissected and collected. And the Total RNAs were collected.

### 2.2 Circular RNA sequencing

Circular RNA samples were sequenced on an Illumina Hiseq. 2500 platform following the manufacture's recommendations. In briefly, libraries were prepared by selecting circular RNAs using Epicentre Ribo-zero™ rRNA Removal Kit (Epicentre, Madison, WI, USA) and RNase R (Epicentre). Subsequently, the qualified libraries were clustered on a cBot Cluster Generation System using HiSeq PE Cluster Kit v4 cBot (Illumina) according to the manufacturer's protocol. After cluster generation, the libraries were sequenced and 150-bp paired-end reads were generated. The full data sets have been submitted to NCBI Sequence Read Archive (SRA) under.

Accession.
