# Supplementary material for: Circular transcriptome sequencing of the middle silk gland and posterior silk gland in the Bombyx mori
Source: Data Brief. 2017 Oct 18;15:709–11. doi: 10.1016/j.dib.2017.10.028 (PMC5671477; doi:10.1016/j.dib.2017.10.028)
Supplement: Supplementary file 1 — Supplementary material [file mmc1.docx]

**'Conflicts of interest: none'**
